# Public service motivation and organizational performance: Catalyzing effects of altruism, perceived social impact and political support

**DOI:** 10.1371/journal.pone.0260559

**Published:** 2021-12-02

**Authors:** Syed Sohaib Zubair, Mukaram Ali Khan, Aamna Tariq Mukaram

**Affiliations:** 1 Department of Administrative Sciences, University of the Punjab, Jhelum, Pakistan; 2 Institute of Administrative Sciences, University of the Punjab, Lahore, Pakistan; 3 Islamia University Bahawalpur, Bahawalpur, Pakistan; Universiti Pertahanan Nasional Malaysia, MALAYSIA

## Abstract

With the increasing pressures and demands from the public sector to be more efficient and effective and accountable, the idea of Public Service Motivation (PSM) and Organization Performance (OP) has become more relevant and critical. This quantitative research hypothesizes that PSM leads towards higher level of organizational performance among public sector officials and also explores the intervening effects of Altruism (ALT), Perceived Social Impact (PSI) and Political Support (PS) in this context. Based on self-administered questionnaire, data was collected from 405 public officials using random sampling strategy. Covariance Based Structural Equation Modelling was used to test the hypothesized model. Following the validation of the measurement model, structural model was developed to test the various paths predicted in the hypotheses. Analysis revealed that PSM, PS and ALT have a positive relationship with OP whereas PSM relationship with PS could not be established.

## 1. Introduction

Governance and Government related issues are becoming increasingly complex and it is the need of the hour to focus on various possible solutions in the light of *“dynamics of modern societies”* [1]. Among various issues, the idea of motivating public sector employees has always been one of the major challenges. The literature in public administration has long endeavored to distinguish the characteristic of public and private administration. Public administration theorists and scholars have incorporated an enormous amount of time in anticipating what motivates public sector employees. Due to the reason that public sector lacks in providing explicit financial incentives to the employees and the fact that government employees look up to a clear and meaningful service, the available research in this realm has been majorly tilted towards non-financial factors [2]. Two vastly researched non-financial factors among these are goal clarity i.e. [3] and public service motivation [4]. The understanding of motivation for individuals working in public organizations is a prerequisite for the management and structure of public sector and for a prosperous provision of public services. Motivation in its general description withholds a stimulus that strengthens, sustains and directs the behavior of individuals, while for public service employees these motivational stimuli are specifically associated with the desire contributing in the social world and serving its citizens [5–7]. Studies such as [8] and [9] clearly presents that Public Service Motivation (PSM) cultivate higher performance in organizations only when managers get the instinct of employees feeling that they can hold a constructive influence on society.

According to pioneer studies including [10] and [11], it is assumed that employees in public sectors carry a motivation and zeal of serving public which is not present in private sector employees. Since the very beginning, public sector has been highlighted as a responsibility, a duty and a calling instead of merely being a job because, these employees are supposed to be motivated by the ethics of serving public in contrast to employees working in private sector organizations. While on the other hand, the rational choice theories of public administration view public administrators as self-interest maximizers not paying credits to those moral responsibilities which are not specifically reflecting any explicit goals and the external rewards associated with their achievement [12]. While many organizational theorists and behavioral scientists have tried to indicate the significance of non-selfish motivational elements such as loyalty, altruism and sense of responsibility in overcoming the most highly reported malpractices in public sector organizations such as self-aggrandizement, free riding and opportunism.

A study has highlighted the failures and challenges of traditional incentives in motivating public sector employees [13]. Moreover, [14] demonstrated the negative impacts of pay for performance on public sector and also depicted that these negative effects are more likely to persist in public when compared to the management of private sector. In short, studies such as [15] suggest that by adopting the practices of private sector may not necessarily lead towards the similar perks and advantages of performance in public sector organizations.

Scholars and practitioners in the field have been active in the process of deepening our understanding of why employees in public sector urge to act more in the favor of common good as compared to private sector employees. The leading theoretical perspective which explains the reason why public employees are more active in serving society is public service motivation [16]. According to [17], public service motivation has been defined as the belief, values and attitudes that go beyond self-interest and organizational interest, that concern the interest of a larger political entity and that motivate individuals to act accordingly whenever appropriate.

The growing volume of research in the domain of public service motivation is the spirit of this study. [18] and [19] report that, beforehand the research on public service motivation has predominantly been conducted in European and American context and Asia has generally being under-researched. The notion to improve the performance of public sector organizations in Pakistan carries equal importance. Since, public sector reforms in the country have specifically endorsed ‘merit-based systems’ and a performance oriented culture which is altogether different from traditional practices that levies growth demands on public sector employees and public organizations in general. The study hypothesizes that public service motivation leads towards higher level of organizational performance among public sector officials because they value organizational results and fate as their own. It contributes to theory and evidence by providing meaningful insights into how public service motivation increases organizational performance amidst the presence of altruism, perceived social impact and political support.

## 2. Literature review and theoretical underpinnings

Over a period of more than two decades, studies such as that of [20] and [21] in public administration research have compiled the need to understand the context of work motivation in public sector organizations. Undeniably, work motivation is a complex subject, and no single theory of motivation can address all the contextual settings of work motivation [22]. The advocates of goal theory i.e. [23] promoted that goal theory is conceivably potentially appropriate in the motivational settings of public sector. This assertion may not be true for the reason that it relies on “personal significance reinforcement” instead of monetarist incentives, rather it is considered convincible due to the vital share it carries into many other motivational techniques.

The motivational explanation presented by goal theory illustrates that variations in the performance of employees are not due to the situation or ability rather due to their diverse performance objectives [24]. Likewise, according to the social cognitive theory, goals do not provide enough explanations to motivate employees to perform, rather these are the discrepancies that individuals shape to compare their actual performance with their desired performance which motivate their behaviors [25]. The outcome of these discrepancies is a feeling of self-disapproval or approval which encourages individuals to perform in a way that increases self-approval.

As per [22], the integration of goal and cognitive theories is practical and significantly important to understand motivation in the domain of public sector. Resultantly, if public sector carries ambiguous goals or some conflicting contextual or procedural constraints, these characteristics put a potential influence on the attitudes of employees which as per social cognitive theories are the keystones of work motivation. The present study contributes to the understanding of public service motivation and its relationship with organizational performance by considering altruism, perceived social impact and political support as possible imminent factors that can significantly influence.

Political environment and its corresponding governmental reforms can be a challenge in the path of stimulating the provision of public service motivation despite of having synchronization between organizational and employees’ values. Since, organizations in public sector are typically engrossed in high bureaucratic systems and political structures where individual service providers work within the confined system of directives, rules and regulations and are accountable to their political heads. This does influence their potential of public service motivation and their abilities to uphold [26]. Policies and political environment carry power to influence the working conditions of service providers [8] and when such policy reforms are perceived by service providers as a source of their work support, motivation can be multiplied rather than being curtailed [7, 27].

Studies such as those by [28] show that employees who carry higher level of public service motivation do take part in social and political activities, and these organizations promote several values associated to their motivation. Similarly [29] reveal that people with an orientation for doing good to authors’ value social impact and are likely to be more helpful in certain public services.

Undertaking an institutional and organizational framework, this study argues that the presence of altruism, perceived social support and political support in public service motivation-Organizational Performance relationship demonstrates exactly how this association unfolds. Finally, the study asserts that the presence of contextual factors such as altruism, perceived social impact and political support as potential mediators can assist the relationship between public service motivation and organizational performance. This discussion leads to the elaboration of key constructs in this study, followed by development of research hypotheses that are to be tested.

### 2.1 Public service motivation

The term public service motivation was first coined by [30] which was further elaborated by Perry and Wise formally and in consequence of it the research in the realm of public service motivation was sprouted. The description of [10] states public service motivation as “*the individual predisposition to respond to motives primarily or uniquely found in public institutions*”(p.368). Moreover, in addition to this description, public service motivation is also portrayed as a general orientation of individuals towards delivering services to people with the intention of doing good for society at large [31].

Research about public service motivation has uprooted rapidly since two decades. [10] elaborate that public service motivation is to influence employees’ behavior in three different manners i.e. (a) as the level of public service motivation escalates, individuals are more oriented towards working in public sector organizations (b) public service motivation is significantly correlated with job performance of employees in public organizations and that (c) public organizations are comprised of higher number of employees having higher degrees of public service motivation and are not necessarily in need for extrinsic incentives to fulfill their motivation. While [17] emphasizes on the altruistic component present in public service motivation and further describes it as the beliefs, values and attitudes that go beyond self-interest and organizational interest, that concern the interest of a larger political entity and that motivate individuals to act accordingly whenever appropriate. Perry et al. [32] argue that due to the blend of altruism, public service motivation has to be considered as a specific type of motivation. According to [33], some normative concerns such as political ideologies are also a part of public service motivation. As per [34] and [35], public service motivation does not only measure motivation in public sector employees, it is rather equally applicable for studying the motivation of volunteer workers. Furthermore, in the perspective of public service motivation, employees’ motivation is slanted towards realizing the importance of goals and services in the public sector because they are a part of some specific public employees and hence get to justify their performance and behaviors accordingly [34].

Public service motivation and its role as an independent variable is of special consideration because of the proposed welcoming outcomes research has found it with. Studies have found public service motivation’s association with individual and organizational performance [36]. As per [37], this relationship has been relatively under researched. Furthermore, [18] in their comprehensive literature review have lately reported 34 studies out of around 300 articles over a span of twenty five years. In that review 21 studies demonstrated a positive relation between public service motivation and performance, while the rest represented assorted or neutral finding.

Public administration scholars advocate that the true spirit of public service-motivated employees resides in serving the abstract notion of public interest through contributing and serving the society at large. It is also reasoned that public service motivation which focuses on societal well-being primarily resonates with “*societal altruism*” [38].

### 2.2 Altruism

Altruism comprises of behaviors a person, a group or an organization takes part in for the sake of providing benefits or to improve the wellness of the beneficiaries. It can also be describes as exhibiting one’s own personal resources to benefit others. It works as an ethical doctrine in which the moral values of an individual’s action are dependent solely on their influence over others regardless of their consequences and outcomes on the individual itself. It is also similar to the concept of formal utilitarianism which advocates maximizing acts which hold good consequences for whole society. Moreover, according to [39], altruism is defined as *“acting on genuinely selfless motives to enhance another’s welfare”*. It suggests that altruism is a special behavior grounded on particular sets of fundamental yet theoretically distinctive motives.

In psychology research the concept of altruistic motivation and altruism are considered to describe the motivational dimension. However, studies such as [40] consider it as an ambiguous psychological terminology and argue that it is important to noticeably explain altruism as a behavior, otherwise it may hold identical meanings as the description of prosocial motivation. In line with this description, the present study undertakes the explanation of altruism in the perspective of [41] i.e. *“evolutionary biology”* which expresses altruism as *“conferring a benefit ‘b’ on the recipient at a cost ‘c’ to the donor”*, this definition explicitly withhold the conceptual basis of altruism and align with the concept of a *behavior* and not of a *motivation*. Through the discussion these narrow differences among motivations and behaviors scholars are more able to reduce the complexities by ultimately steering towards conceptual clarity [42]. As per [10] altruism contributes in building normative and affective motives among individuals i.e. the normative aspiration of serving and working for the public interest can be regarded as being altruistic. Scholars such as [43] studied the potential connection of the affective dimension of altruism and selflessness. Piatak and Holt [44] comprehensively describe that public service motivation and altruism undoubtedly measure some intersecting fragments of prosocial motives for behavior but on the other hand they are different concepts where public service motivation is founded to be more likely predicting voluntary behaviors as compared to altruism.

### 2.3 Perceived social impact

The concept of perceived social impact is described in terms of degree to which employees analyze their actions while positively influencing their recipients, for instance, by offering such services and products that create a positive impact in the lives of customers [45, 46]. In some of the pioneer research, the connection between perceived social impact and job performance has been demonstrated clearly. Grant in a series of experiments [45, 47, 48] demonstrated that connection with recipients amplified social impact’s perception and consequently instigated higher persistence and improved work performance.

In a study on public sanitation department, [49] have concluded that perceived social impact significantly curtails emotional collapse and increases administrative performance ratings among employees.

### 2.4 Political support

Easton [50] (p.436) describe political support as the *“degree to which individuals evaluate political objects positively*, *that is*, *the mix of attitudes about political leaders*, *institutions and the system as a whole”*. According to [51–53] there are different faces of political support. Tausendpfund and Schäfer [54] distinguishes “overt support”, that are “supportive activities”, such as vote casting in favor of some political candidate and “covert support”, that is associated with “supportive behaviors” i.e. party loyalty. Moreover, according to [55] the concept of political support acts as multidimensional because it includes contentment with policies as well as a general assessment which reports how well a political system, its authorities or institutions are meeting the normative expectations of its residents. As per [56] and [57], political support elevates in the presence of direct democratic instruments which are considered while political decision making. Moreover, with reference to the procedural fairness theory, [58] argues that just procedures curtail the negative consequences of unsuitable decisions, which means that citizens may not receive the desired outcome but since, they held a support for raising their voice in the processes, they endorse the processes and call them just and fair which in consequence amplify their political support.

Furthermore, Bowler and Donovan [59] (p.376) explains that citizens due to the notion of direct and democratic decision-making hold an “occasional voice in government”, which means that their voices are given a considerable attention and they are able to take decisions on specific issues and are listened to. This notion of feeling themselves as a credible part of decision making signifies their perception of influence and political support. According to [60] this practice largely illustrates their sense of self-determination along with a significant sense of control on their society and living conditions. Shomer et al. [61] illustrates that the higher degrees of people’s involvement and participation in electoral procedures for the political parties amplifies political support.

### 2.5 Organizational performance

**Fig 1:**
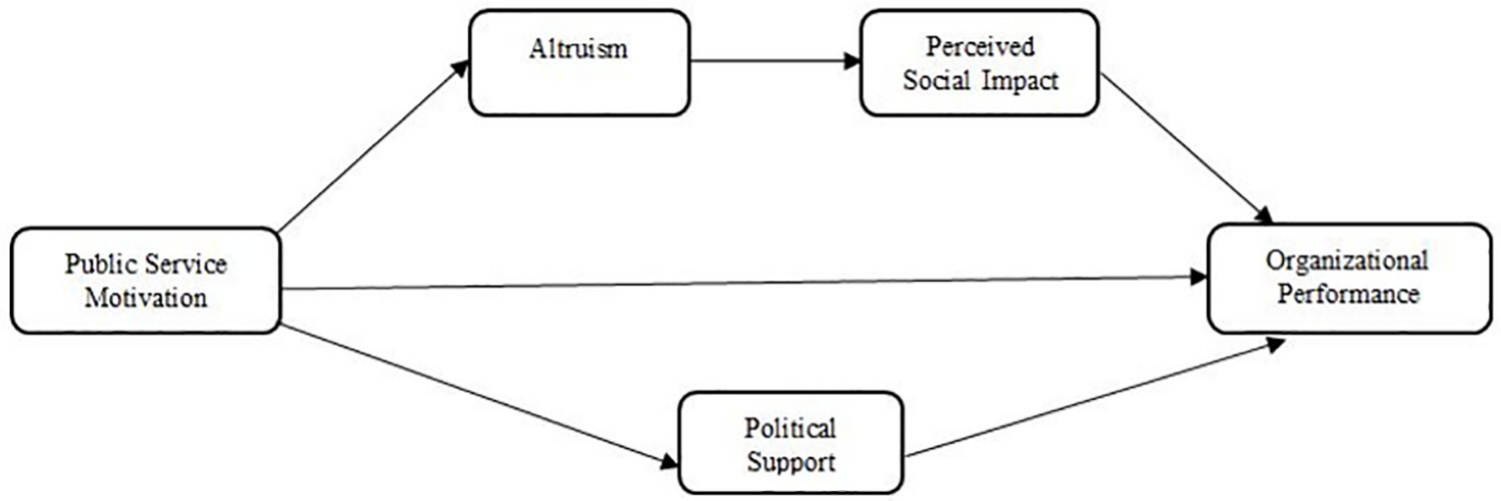
Research model.

Organizational performance is generally theorized in terms of the actual output of an organization which are measured against its desired or intended results, objectives or goals and meet the expectations of different groups of stakeholders [62]. The level of organizational performance is evaluated through several elements consisting of operational efficiencies, levels of diversification, mergers, acquisitions, composition of top management and organizational structures and manipulation of social or political effects interfering with the market conformity [63]. Although, the measuring criteria for organizational performance has been remained controversial. Studies such as [64] endorse adopting a multi-dimensional approach to measure organizational performance which reflect a broader range of interests of stakeholders. However, Rouse and Putterill [65] demonstrates that there is no single performance criteria that is suffice enough to be applicable for all organizations. Hence, organizational performance being a complex subject should always be studied in the contextual settings of the existing context [66]. Exceptional results are maintained by organizations when they meet the expectations of stakeholders within society [67]. Based on all this discussion and the objectives of the study, Fig 1 below depicts the research model developed for the study.

### 2.6 Hypothesis development

Distinct studies such as [68, 69] illustrate that public service motivation leads towards individual performance. [17] validates the potential evidence that public service motivation is positively connected with job performance. Moreover, an empirical study conducted on medical staff i.e. nurses in Italy proliferates that public service motivation carries a significant positive association with performance [70]. In some relative studies comprising of small samples from nurses, school teachers and other government employees represented a positive relationship of public service motivation and job performance i.e. [70, 71, 36].

Public administration scholars and experts are captivated in knowing the way PSM amplifies organizational performance of employees in public sector organizations [37]. In the available literature PSM has been associated to primarily positive consequences, such as organizational citizenship behavior as depicted by [72], organizational commitment as studied by [73] and job satisfaction as portrayed by [74]. However, there is a lack of empirical research on the relationship between public service motivation and organizational performance and this relationship is still inconclusive in the available literature [37].

In addition to this discussion, [75] describes that the conceptuality of performance and what creates performance in public sector is complex because it might comprises of some private sector measures such as efficiency or it may carry orientation towards more of public setting objectives such as transparency, access to public, and alleviation of corruption. A meta-analysis [76] demonstrated that a performance surge and a higher possibility of better performance can be seen with the help of intrinsic motivational sources in contrast with extrinsic motivators. According to [77], there is a significant positive connection between public service motivation and organizational performance. In the light of these findings this study leads towards hypothesizing that public service motivation is potentially related with organizational performance.

**H1: Public service motivation is significantly positively related with organizational performance.**

As far as the relationship between public service motivation and political support is concerned, there are quite a few studies which have been conducted on said variables. According to a research conducted on undergraduate students public service motivation is identified as one of the major factors in increasing political participation and support [78]. Another study conducted on 300 civil servants found a positive and significant relationship between political support/loyalty and public service motivation [79].

As far as relationship between political support and organizational performance is concerned, a study conducted by [80] highlighted the positive role of organizational performance in unfolding the role of political support and concluded that political support is inevitable in accessing organizational performance. According to [81], a study conducted on elected officials found a positive relationship between political support and organizational performance. Based on following studies, following hypotheses have been developed;

**H2: Public service motivation holds a significant positive relationship with political support.**
**H2a: Political support is significantly and positively associated with organizational performance.**
**H2b: Political support performs as a potential mediator between PSM and organizational performance.**

As far as relationship between public service motivation and Altruism is concerned, there are very few studies which are conducted on the relationship between these two concepts as number of studies tried to distinguish these two concepts [42, 44]. As per [82], a late study conducted in 1870 on university students resulted into finding that public service motivation and altruism are significantly positively correlated with each other and also found that public service motivation may act as a potential predictor of Altruism.

There are quite few studies steered on trying to develop the connection between Altruism and social impact. According to [83], it was concluded that there is a crucial role of altruism in the society which can eventually create positive social impact. According to [84], a study conducted on US public and non-profit employees concludes that performance metrics are more likely to be used by those public servants who consider social impact as an important aspect of their tasks. According to [46], study concluded the positive relationship between perceived social impact and performance. Based on the aforementioned discussion, subsequent hypotheses are developed;

**H3: Public service motivation is significantly positively associated with altruism.**
**H3a: Altruism is expected to have a significant positive relationship with perceived social impact.**
**H3b: Perceived social impact is anticipated to be positively associated with organizational performance.**
**H3c: Altruism and perceived social impact mediates the relationship of PSM and organizational performance.**

## 3. Methodology

Design and protocols developed or followed for a study are of critical nature [85]. They add that no matter how advanced statistical tool a researcher uses, the research effort might not have sound weightage if the fundamentals of research design and methodology are not carefully taken care off. This research on various factors associated with public officials and their performance has been evaluated by following a quantitative research methodology and a cross-sectional research design. The sample included officers from public organizations/departments under the federal and provincial governments in Pakistan, where the population is 1343, as per the list available with the central bank i.e. the State Bank of Pakistan. On the basis of [86], the minimum sample size calculated was to be 308. The questionnaires were sent to 475 civil servants using random sampling strategy and 405 were received as duly filled making the response rate of approximately 85.26%. The reason for sending 475 questionnaires was the potential issue of no response, however, the response rate was good in actual. The said individuals in the sample representing their organizations were from top-tier management. In order to tap the organizations, simple random sampling strategy was used and organizations were selected from the frame available. It is imperative to mention that verbal informed consent was taken from the respondents and all details regarding the purpose of data collection and the research work were shared in a cover letter attached with the instrument.

Furthermore, in order to collect data, a structured questionnaire was adapted after extensive review of the literature and responses were recorded using a 5 point Likert Scale. Table 1 highlights the scale used to measure all variables, the number of items used and a sample item for each construct:

**Table 1:** Measures.

Latent Construct	Source	Number of Items	Sample Item
Public Service Motivation	[11, 87]	05	Making a difference in society means more to me than personal achievements
Altruism	[88]	04	I have sympathy for people who are less fortunate than I am
Perceived Social Impact	[45, 89]	04	I feel that my work makes a positive difference in other people’s lives
Political Support	[90]	03	Most elected officials believe that our organization is effective
Organizational Performance	[91]	08	My organization is trying to reduce cost in managing organization and performing works

The model developed earlier, and the collected valuable responses were put to inferential analysis using Co-Variance Based Structural Equation Modelling (SEM) through AMOS. Prior to the testing of the hypothesis through the structural model, several perquisites were established and ensured using the confirmatory factor analysis (CFA). The model fitness was tested and ensured, followed by the confirmation of the convergent and discriminant validities. Table 2 summarizes the demographical characteristics of the officers that were part of the final sample:

**Table 2:** Sample characteristics.

Category	Items	Frequency	Percentage
**Gender**			
	Male	340	84
	Female	65	16
**Years of Service**			
	Less than 15 Years	136	33.6
	More than 15 Years	269	66.4
**Education**			
	Bachelors	135	33.3
	Masters/M.Phil	259	64
	PhD	11	2.7
**Department/Office Currently Serving**			
	Federal	164	40.5
	Provincial	199	49.1
	District	42	10.4

The sample included 84% male officers and 16% female officers playing a lead role in the organizations under study. 33.6% of the total sample was officers with experience of less than 15 years whereas 66.4% were of more than 15 years of service in the public sector. Referring to the education of such officers, 33.3% were bachelors, 64% had a Masters/M.Phil degree whereas 2.7% had a PhD. Public organizations or offices in Pakistan range from federal, provincial and district level. 40.5% respondents were from federal organizations, 49.1% from provincial organizations, whereas 10.4% were currently serving in district level organizations.

## 4. Results

This study has used descriptive statistics including the Means and Standard Deviations of the latent constructs whereas measurement and structural model using covariance based SEM. As far as descriptives are concerned, the mean values of all latent constructs are between 2.74 and 3.68 whereas standard deviations are from 0.54 to 0.81 shows the dispersion of mean.

### 4.1 Measurement model

The purpose of measurement model is to check the reliability and validity of the model. It also identifies the model fitness indices which ultimately decide the fitness of the model. At first stage, it is highlighted that the Standardized Factor Loading (SFL) of each item should be at least 0.60. However, as per the initial model, the only item whose factor loading was found to be less than the threshold value was PSM 1. After removing the said item, the model was run again and found all the items more than the threshold value of 0.60. At first stage, model fitness indices were tested using covariance based Structural Equation Modeling (SEM). As far as relative chi-square value is concerned, its threshold value is up to 3 [92] which stands true in this case as the value was found to be 2.90. Moving on, Goodness of Fit index (GFI) [93], Normed Fit Index (NFI) [94], and Tucker Lewis index (TLI) [95] have threshold values of minimum of 0.90 and in this case, all values meets the minimum threshold with the value i.e. 0.901, 0.927 and 0.941 respectively. Furthermore, Comparative Fit Index (CFI) minimum threshold is 0.940 [96] and its obtained value is 0.950. Lastly, RMSEA minimum threshold is up to 0.080 and in this case, it is 0.069 meeting the minimum threshold [97].

#### 4.1.1 Composite reliability and convergent validity

Table 3 highlights the composite reliability and convergent validity. Convergent validity which refers to the accuracy of convergence of items towards their respective latent constructs [98]. For fulfilling the criteria of convergent validity, three criteria must be fulfilled. One the minimum SFLs must be at least 0.60 which is the case in this study. Secondly, Composite reliability (CR) refers to the internal consistency of the items and its values should be at least 0.70 [99] which in this case stands true as CR of public service motivation, altruism, social impact, political support, and organizational performance is 0.826, 0.838, 0.854, 0.820 and 0.939 respectively. Thirdly, Average Variance Extracted (AVE) should be at least 0.50 [97] which also stands true in this case as AVE of public service motivation, altruism, social impact, political support, and organizational performance is 0.544, 0.567, 0.593, 0.604 and 0.660 respectively. Looking at the aforementioned discussion, it is concluded that convergent validity exist in the model.

**Table 3:** Composite reliability and convergent validity.

Latent Construct	Items	SFL	CR	AVE
Public Service Motivation	PSM2	0.731	0.826	0.544
	PSM3	0.801		
	PSM4	0.676		
	PSM5	0.736		
Altruism	ALT1	0.793	0.838	0.567
	ALT2	0.816		
	ALT3	0.748		
	ALT4	0.642		
Perceived Social Impact	PSI1	0.784	0.854	0.593
	PSI2	0.773		
	PSI3	0.784		
	PSI4	0.738		
Political Support	PS1	0.708	0.820	0.604
	PS2	0.820		
	PS3	0.799		
Organizational Performance Political Support	OP1	0.747	0.939	0.660
	OP2	0.700		
	OP3	0.857		
	OP4	0.819		
	OP5	0.866		
	OP6	0.839		
	OP7	0.799		
	OP8	0.855		

#### 4.1.2 Discriminant validity

As far as discriminant validity is concerned, it refers to the level to which participants were able to differentiate between the items of latent constructs [97]. For meeting the criteria, all the values of the correlations should be less than the square roots of AVEs. As per Table 4, it can be seen that all the values of the correlations are less than the square roots of AVE which means that discriminant validity exist in the model.

**Table 4:** Discriminant validity.

	PSM	ALT	PSI	PS	OP
PSM	0.737*				
ALT	0.352	0.753*			
PSI	0.021	0.393	0.770*		
PS	0.031	0.243	0.342	0.777*	
OP	0.412	0.014	0.404	0.374	0.812*

Lastly, as far as Common method Bias (CMB) is concerned, “Harman Single Factor Test” (HSFT) is used which is referred to see whether “change in single factor affects all the variables in the data and that variance should be less than 0.5 to avoid CMB” and in this study, value of HSFT is found to be 0.09, therefore it is reported that data is not suffering from CMB [100]. However, there are few limitations associated with technique [101], hence, “Common Latent Factor” (CLF) test is used through SEM by “comparing standardized regression weights (SRWs) with and without CLF and found that SRWs without CLF were higher than SRWs with CLF with the difference of less than 0.05”, ultimately concludes that data is not having CMB [102].

### 4.2 Structural model

**Fig 2:**
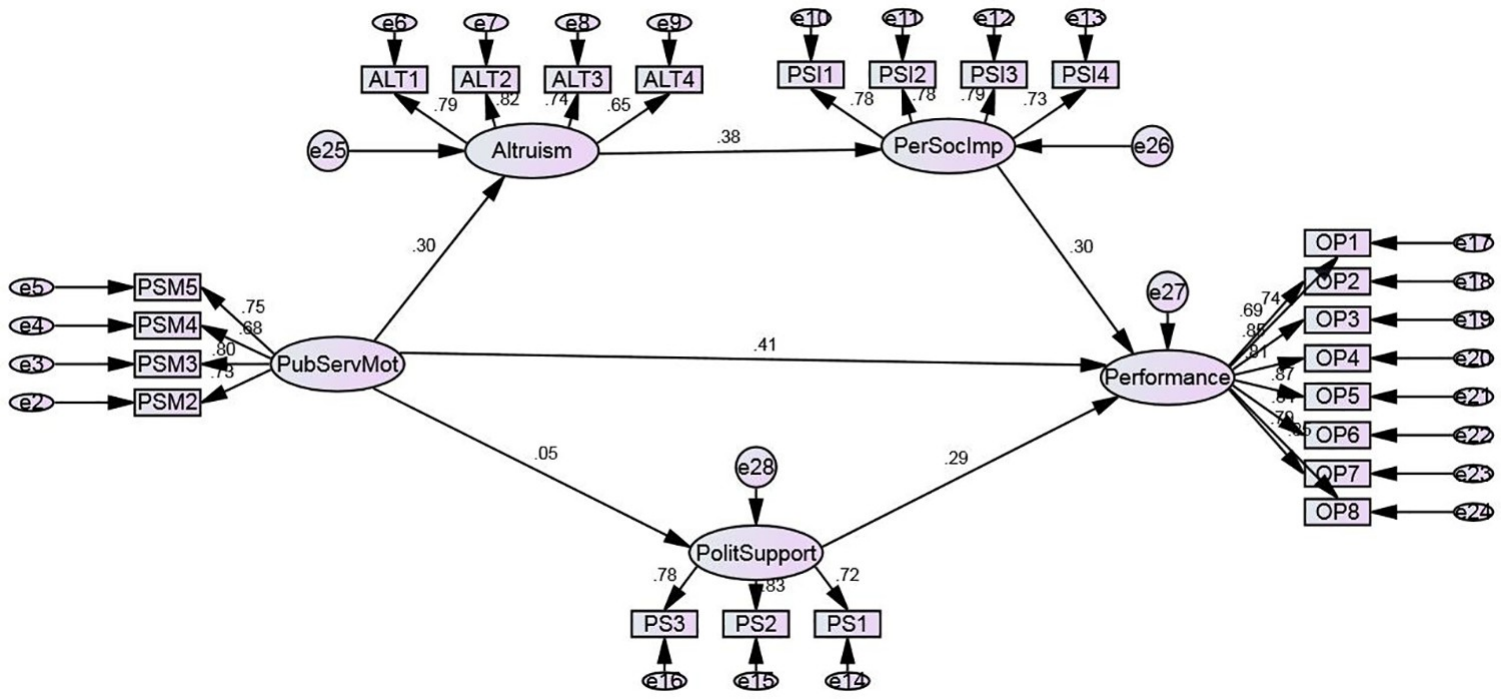
Structural model.

Fig 2 is the structural model developed for testing the hypotheses of the study. As per Table 5, it can be seen that public service motivation is directly and positively related to organizational performance at β = 0.41 which approves first hypothesis. As far as public service motivation relationship with political support is concerned, the relationship was not found to be significant at β = 0.05 and rejected second hypothesis. As far as political support relationship with organizational performance is concerned, it was found to be significantly positive at β = 0.29 and approved H2a. Due to the rejection of H2, mediation path due to political support between public service motivation and organizational performance was also found to be insignificant at β = 0.015. As far as relationship between public service motivation and altruism is concerned, the relationship was found to be significantly positive at β = 0.30 leading to the acceptance of H3. Similar relationship was found between altruism and social impact and social impact and organizational performance at β = 0.38 and β = 0.30 accepting the H3a and H3b. Due to these significant relationships, serial mediation due to altruism and social impact between public service motivation and organizational performance was found to be significant at β = 0.034 approving H3c and similar results were found by taking altruism as mediator between public service motivation and social impact at β = 0.11 leading to the acceptance of H3d and by taking social impact as mediator between altruism and organizational performance at β = 0.41 ultimately the acceptance of H3d.

**Table 5:** Direct and indirect effects.

**Direct Effects**		
	**Path Coefficients**	**Status**
H1: PSM → OP	0.41**	Accepted
H2: PSM → PS	0.05	Rejected
H2a: PS → OP	0.29**	Accepted
H3: PSM → ALT	0.30**	Accepted
H3a: ALT → PSI	0.38**	Accepted
H3b: PSI → OP	0.30**	Accepted
**Indirect Effects**		
H2b: PSM → PS → OP	0.015	Rejected
H3c: PSM → ALT → PSI →OP	0.034*	Accepted
H3d: PSM → ALT → PSI	0.11**	Accepted
H3e: ALT → PSI →OP	0.11**	Accepted

**Significant at 1%.

*Significant at 5%.

## 5. Discussion and conclusion

This study aims to respond the recent for empirical research into the association between public service motivation and organizational performance. The relationship between public service motivation and organizational performance carries an utmost significance for researchers’ community because scholars are eager in identifying some predictable connection between what motivates employees and stems their organizational performance in the public sector. It is direly important to look into these concepts and strengthened them owing to the high stakes involved in the public sector.

Results found that public service motivation is significantly and positively related with organizational performance as reflected in H_1_. The pioneer of the idea of public service motivation i.e. [10] argued that employees having greater level of public service motivation carry greater chances of performing better in public sector organizations. The positive insights regarding the relationship between public service motivation and organizational performance supports a recent empirical study [70] in this domain.

Moreover, two highly cited studies i.e. [103] and [104] based on sectoral comparison reported that employees from public sector held a greater enthusiasm towards helping others and working for societal benefits. Moreover, the results from public and private sectors confirmed that public sector employees are more altruistic in their behaviours and are more prosocial as compared to other members of society. Likewise, [73] examined a significant positive relationship of public service motivation with performance and support for governmental reinvention activities.

Furthermore, [105] elaborate two premises in this domain. The first premise describes that public service motivation is more like a behavioral trail where altruistic characteristic motivates prosocial behavior among employees. The second premise holds description that people in every walk of life can have altruistic traits and be motivated to perform social service, public service motivation is more likely to be present in public sector employees as compared to private sector and elsewhere.

Moreover, the findings support the serial mediation path in the conceptual model (H3c) i.e. PSM → ALT → PSI →OP which hypothesizes that altruism and perceived social impact mediates the relationship of public service motivation and organizational performance. The results also suggest that public service motivation is strongly and positively related with altruism hence, approving the assumption of H3. On the basis of similarity between public service motivation and altruism some scholars encourage to establish some conceptual boundaries between them [18]. Scholars such as [21] have used public service motivation as some general interchangeable concept of altruism. While others have distinguished public service motivation as a prosocial motivational element that is primarily grounded in public sector employees and altruism as a general motivational dimension which aids to serve more specific subgroups among people. Scholars also agree that altruism is one the multiple dimensions of public service motivation [32, 43, 106]. Public service motivation is more likely understood as a general motivation directed towards society or individuals; it is highly expected that public service motivated employees indulge in different types of altruistic behaviors or societal altruism. Moreover, it is argued that public service motivation which potentially directs towards society is associated with societal altruism. The results of this study which show that public service motivation is positively associated with altruism, which are in line with [9, 38, 42].

Moreover, the results indicated that altruism is positively related with perceived social impact and validated the postulation of H3a. In relation with these findings [107] suggest that public service motivation potentially predicts employees’ perception of social impact of their jobs. Moreover, [45] showed that employees’ motivation can be amplified when linked with the prosocial impact of their jobs.

In addition to this, the last path of serial mediation approves that perceived social impact is significantly and positively related with organizational performance hence supporting H3b. The results equate with [45] which found that perceived social impact brings about dedication and is positively related with performance. Furthermore, [46] describes that perceived social impact plays a positive role in determining employees’ motivation to perform their jobs well. Existing empirical research in this realm such as [17, 74, 78] provide evidence that the real benefits of public service motivation may rely on employees’ perception that their work provides them with enough opportunities to serve others. Moreover, [49] and [108] argue that higher degrees of perceived social impact lower emotional exhaustion of employees and stimulate them towards higher performance. [84] present that when public sector employees are pro-socially motivated and perceive a meaningful influence and purpose of their job on others, they provide organization with high end performance gifts.

The data did not show support for the overall mediation path i.e. H2b which hypothesized that political support performs as a potential mediator between public service motivation and organizational performance.

Noticeably, the results did not validate the assumption of path A of mediation i.e. H2 which hypothesized that public service motivation is positively related with political support. [109] support the findings by illuminating that public sector employees having higher levels of public service motivation are more vulnerable to perceptions of politics as compared to those having lower levels of public service motivation. In addition, [110] emphasize that public sector employees carry higher levels of self-efficacy and can be more productive when they perceive their organization to be less political or non-political. Keeping this view it can be assumed that public service motivation is a behavioral trait and public service motivated employees are not necessarily reliant or in wait for political support in their respective organizations.

While, the path B of mediation i.e. H2a which postulated that political support is positively related with organizational performance was supported by the data. It is normally argued that the firms which bear high political support carry easy access ability towards long term governmental loans and other governmental privileges. The findings of this study equate with [111–114] and suggest that being politically supported ultimately upsurges organizational ability to showcase higher performance. In addition to this, [112] demonstrates the importance of political regimes by approving that the performance of politically supported organizations in Pakistan increased during political regimes when compared with military regimes.

The study generates enough evidence that the presence of public service motivation carries a positive impact on employees’ job behavior and organizational performance in particular. It is therefore inevitable for public sector organizations to seek ways to maximize and encourage public service motivation among their employees. It concludes that altruism and perceived social impact positively mediates the association of public service motivation and organizational performance. While political support does not validate itself as a potential mediator between public service motivation and organizational performance. However, political support individually proves itself to be a potential predictor of organizational performance. To sum it up, Public Service Motivation is a concept that is not just of scholarly interest to academicians but it equally interests and applies to practitioners particularly public administrators and managers that need to deal with multiple complexities and challenges, varying from efficient use of financial and human resources in order to make sure that the public offices and organizations are responsive to the public, and meeting its objectives [115].

### 5.1 Managerial implications

The present study provides relevant insights and practical implications for public sector organizations, their employees and managers by adding its valuable evidence which supports the role of public service motivation and its contribution in achieving organizational performance. It provides a meaningful contribution by providing a practical usefulness of undertaken constructs i.e. public service motivation, organizational performance, social support and political support in the field of research in public administration. The observed relationship between public service motivation and organizational performance can be useful in measuring the behavioral traits and channeling the performance and motivation of public sector employees. Moreover, the findings are useful for practitioners because they demonstrate the importance of employees’ perceptions of social impact and emphasize their positive role in relation with organizational performance. It is reiterated that organizational performance in the context of public sector are very crucial, owing to the fact that high stakes involved and increasing demand for efficiency and effectiveness along with the demand for accountability. Therefore, the model developed in this study syncs with the emerging requirements of the global public sector.

### 5.2 Limitations and future directions

The study acknowledges few limitations. First, the cross sectional nature of the study limits it to assert the possibility of causation among variables. Another possible threat is related to the validity and truthfulness of employees’ belief and the reliance on them because, they cannot be observed or measured directly such as public service motivation and perceived social impact. An earlier research i.e. [116] found that diverging personality traits may influence research related to such concepts. Hence, an inability and limitation to control some personality traits such as altruism, public service motivation or perceived social impact always prevail in such research. Furthermore, demographic factors have not been controlled in this study making it as one of the limitations. Moreover, the generalizability of these empirical findings is limited since, it comprises the contextual settings of public sector organizations in Pakistan, however some findings may be attributed to the developing countries with a similar political and administrative infrastructure.

Future research may introduce a longitudinal research design to study the influence of time lag between the exogenous i.e. public service motivation; mediators i.e. altruism, perceived social impact and political support; and endogenous variable i.e. organizational performance. Furthermore, a multilevel analysis with data from affectees of certain public sector organizations can enrich the literature and provide further insights.

## Supporting information

S1 Data (SAV)
